# Defect of Mitotic Vimentin Phosphorylation Causes Microophthalmia and Cataract via Aneuploidy and Senescence in Lens Epithelial Cells*^♦^

**DOI:** 10.1074/jbc.M113.514737

**Published:** 2013-10-18

**Authors:** Makoto Matsuyama, Hiroki Tanaka, Akihito Inoko, Hidemasa Goto, Shigenobu Yonemura, Kyoko Kobori, Yuko Hayashi, Eisaku Kondo, Shigeyoshi Itohara, Ichiro Izawa, Masaki Inagaki

**Affiliations:** From the Divisions of ‡ Biochemistry and; ‖ Oncological Pathology, Aichi Cancer Center Research Institute, Nagoya, Aichi 464-8681,; the Departments of § Cellular Oncology and; ** Epidemiology, Nagoya University Graduate School of Medicine, Nagoya, Aichi 466-8550,; the ¶ Electron Microscope Laboratory, RIKEN Center for Developmental Biology, Kobe, Hyogo 650-0047, and; the ‡‡ Laboratory for Behavioral Genetics, RIKEN Brain Science Institute, 2-1 Hirosawa, Wako 351-0198, Japan

**Keywords:** Cataract, Cell Division, Cytokinesis, Mitosis, Phosphorylation, Senescence, Aneuploidy, Chromosomal Instability, Vimentin

## Abstract

Vimentin, a type III intermediate filament (IF) protein, is phosphorylated predominantly in mitosis. The expression of a phosphorylation-compromised vimentin mutant in T24 cultured cells leads to cytokinetic failure, resulting in binucleation (multinucleation). The physiological significance of intermediate filament phosphorylation during mitosis for organogenesis and tissue homeostasis was uncertain. Here, we generated knock-in mice expressing vimentin that have had the serine sites phosphorylated during mitosis substituted by alanine residues. Homozygotic mice (*VIM^SA/SA^*) presented with microophthalmia and cataracts in the lens, whereas heterozygotic mice (*VIM^WT/SA^*) were indistinguishable from WT (*VIM^WT/WT^*) mice. In *VIM^SA/SA^* mice, lens epithelial cell number was not only reduced but the cells also exhibited chromosomal instability, including binucleation and aneuploidy. Electron microscopy revealed fiber membranes that were disorganized in the lenses of *VIM^SA/SA^*, reminiscent of similar characteristic changes seen in age-related cataracts. Because the mRNA level of the senescence (aging)-related gene was significantly elevated in samples from *VIM^SA/SA^*, the lens phenotype suggests a possible causal relationship between chromosomal instability and premature aging.

## Teaser

**Background:** Vimentin, an intermediate filament (IF) protein, is phosphorylated in mitosis.

**Results:** Disruption of vimentin phosphorylation during cell division leads to chromosomal instability (CIN) and premature aging in mouse lens tissue.

**Conclusion:** Our data document the first physiological importance of vimentin phosphorylation during mitosis for organogenesis and tissue homeostasis.

**Significance:** Our data suggest a possible causal relationship between CIN and premature aging.

## Introduction

Intermediate filaments (IFs),^3^ together with microtubules and actin filaments, form the cytoskeletal framework in the cytoplasm of various eukaryotic cells. IF proteins are divided into six groups related to their primary sequence characteristics, their tissue-specific expression profiles, and differentiation status (1–3). For example, GFAP and desmin are type III IF proteins that are expressed specifically in astroglial and muscular cells, respectively (4–6). However, vimentin, another type III IF protein, is expressed in all mesenchymal cells with the eye lens being the tissue with by far the highest levels (7, 8).

There is increasing evidence that IF disassembly is regulated by phosphorylation of Ser/Thr residues in the amino-terminal head domain on IF proteins (9, 10). The first direct evidence was obtained by an *in vitro* study that treated polymerized vimentin filaments with a purified protein kinase to cause their disassembly (11). Site- and phosphorylation state-specific antibodies that can recognize a phosphorylated residue and its flanking sequence (12, 13) are powerful tools able to resolve the spatial and temporal details of IF phosphorylation in cells (14, 15). Using antibodies raised against four distinct phosphorylation sites in GFAP, we showed that mitotic IF phosphorylation is regulated by different protein kinases in a spatiotemporal manner (13, 16). Further detailed studies revealed that several mitotic kinases, such as Aurora-B (17, 18), Cdk1 (19, 20), Plk1 (21), and Rho kinase (22–24), participate in the phosphorylation of type III IF proteins during cell division. By transient expression of type III IF proteins mutated at these mitotic phosphorylation sites to Ala, we found that preventing the phosphorylation of IFs during cell division inhibited cytokinesis by the retention of an IF bridge that connected the two daughter cells (17, 18, 21, 25, 26). These findings indicated that mitotic IF phosphorylation is essential for the efficient separation of the two daughter cells. The presence of the IF bridge presented the cell pair with two options. In the first option, the IF bridge could be torn apart presumably by cell adhesion-dependent traction force (27) to allow the completion of cytokinesis (26). In the second option, cytokinesis failed, resulting in the formation of binucleate (multinucleate) cells (21). The physiological significance of IF phosphorylation during cell division for organogenesis and tissue homeostasis, however, has yet to be determined.

**FIGURE 1.**
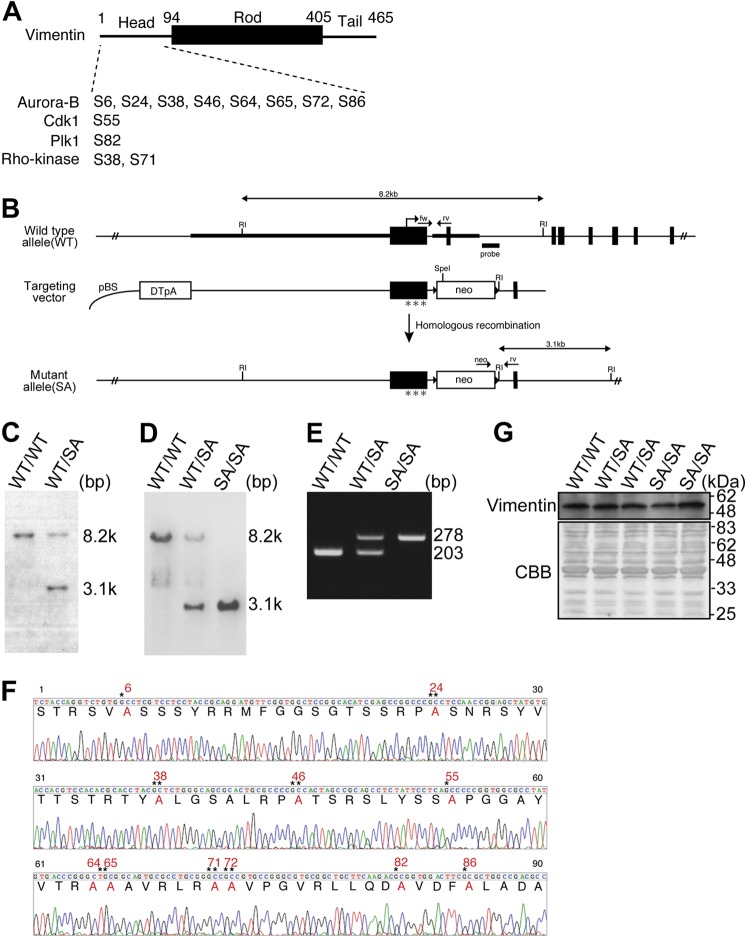
**Generation of knock-in mice (*VIM^SA/SA^*) where two *VIM* alleles are mutated at mitosis-specific phosphorylation sites to Ala.** *A*, schematic featuring mitosis-specific vimentin phosphorylation sites on mouse vimentin. Their responding kinases are also shown. *B*, diagram of the *VIM* genomic locus is shown as follows: WT allele with EcoRI (*RI*) restriction maps, exons (*black box*), and translation initiation point (*hooked arrow*); targeting vector, including mutated exon1 (with *asterisks*), and inserting *neo* gene; mutant allele by homologous recombination. Genotyping probes for Southern blot (*bar*) and the PCR primers (*arrows*) are also illustrated. *C* and *D,* Southern blotting of EcoRI-digested genomic DNA from ES clones (*C*) and tails form offspring (*D*). About 8.2- or 3.1-kbp fragment is derived from EcoRI-digested genomic DNA of a *VIM^WT^* or *neo*-inserted *VIM^SA^* allele, respectively. *E,* PCR genotyping of offspring. The 278- or 203-bp fragment is derived from mRNA of a *VIM^SA^* or *VIM^WT^* allele, respectively. *F,* sequence of mutation sites on *VIM^SA/SA^* were checked as described under “Experimental Procedures.” *G,* Western blot analyses with extracts from embryos. After blotting, the membrane was stained with Coomassie Brilliant Blue (*CBB*). *, *p* < 0.05; **, *p* < 0.01; ***, *p* < 0.001.

We have generated knock-in mice to produce *VIM* alleles in which the serine residues in vimentin that are phosphorylated in mitosis have been replaced with alanine residues (indicated in Fig. 1*A*). Homozygotic mice bearing such mutations (*VIM^SA/SA^*) presented with microophthalmia and lens cataract as phenotypes. The heterozygotes (*VIM^WT/SA^*) showed no obvious phenotype. Compared with WT (*VIM^WT/WT^*) and *VIM^WT/SA^* mice, the epithelial cell number was significantly reduced in lenses from *VIM^SA/SA^* mice. We observed not only binucleate (multinucleate) cells but also aneuploid cells in *VIM^SA/SA^* lens, whereas these abnormal cells were not detected in *VIM^WT/WT^* or *VIM^WT/SA^* lens. In addition, the mRNA level of the senescence (aging)-related gene was significantly elevated in the lens of *VIM^SA/SA^*.

## EXPERIMENTAL PROCEDURES

### 

#### 

##### Generation of Vimentin Knock-in Mice

A bacterial artificial chromosome clone containing the 129Sv mouse vimentin genome locus was purchased (Mouse bMQ bacterial artificial chromosome library, The Wellcome Trust Sanger Institute). The targeting vector was designed to substitute Ser to Ala in the vimentin head domain at the indicated positions (Fig. 1, *A* and *B*). The 5′ and 3′ homology arms were amplified by PCR. Site-directed mutagenesis was introduced on exon 1 for the substitution. Then they were ligated into the 5′ and 3′ side of the PGKneobpA-loxP-positive selection marker cassettes, with an MC1DTpA-negative selection marker cassette at the 5′ homologous arm to enrich for homologous recombinants. The targeting vector was electroporated into EB3 ES cells with 129 background, as described previously (28, 29).

G418-resistant clones were initially screened by Southern blotting analysis with a unique external probe following EcoRI digestion (see Fig. 1, *B* and *C*). Correctly targeted ES clones were microinjected into C57BL/6 blastocysts, which were in turn transferred into foster mothers to obtain chimeric mice. Following germ line transmission, homozygous mutant (*VIM^SA/SA^*) mice were produced by intercrossing heterozygous (*VIM^WT/SA^*) mice (Fig. 1, *D* and *E*). Mutations were verified by PCR followed by DNA sequencing (Fig. 1*F*). The mutant mice were backcrossed for more than 10 generations onto a C57BL/6 background. Genotyping was performed by PCR (Fig. 1*E*) with the following primers: WT (5′-GAT CAG CTC ACC AAC GAC AAG-3′), reverse (5′-TCC TCT GCT ATC CTC CAG ACA-3′), and bpA (5′-TGC ATC GCA TTG TCT GAG TAG-3′). Mice were maintained at the Aichi Cancer Center Research Institute Animal Facility in compliance with the regulations of the Animal Ethics and Animal Care Committees at the Aichi Cancer Center.

##### Immunoblotting

Samples preparation was described previously (30). After incubation with the desired antibody, PVDF membranes (Immobilon-P, Millipore) were developed using the following detection reagents (Western Lightning Chemiluminescence Reagent plus, PerkinElmer Life Sciences, or SuperSignal West Femto Maximum Sensitivity Substrate, or Thermo Fisher Scientific).

##### In Situ Hybridization

*In situ* hybridization on whole-mount embryos was performed using digoxigenin-labeled riboprobes as described (31). Templates were cloned from cDNA by PCR amplification with the following primers: vimentin, 5′-atgtctaccaggtctgtg-3′ and 5′-cgcacatcacgcagggca-3′, and desmin, 5′-cgaggctacacagcaaca-3′ and 5′-tgcctctctcttccttcctct-3′.

##### Histology and Immunohistochemistry

Fixation and H&E staining of histological sections was performed according to standard protocols. For immunohistochemistry, 3,3′-diaminobenzidine staining was performed with a kit (Dako EnVision+System-HRP Labeled Polymer, Dako), according to the manufacturer's protocol.

##### Immunostaining

Paraformaldehyde-fixed tissue slides were deparaffinized, microwaved in 10 mm citrate buffer, pH 6.0, for 15 min, and then blocked in 5% (v/v) donkey serum in TBST for 1 h. Sections were incubated with primary antibodies overnight at 4 °C followed by TBST wash and then incubated with appropriate secondary antibodies (Invitrogen) for 1 h at room temperature.

Cultured cells were grown on coverslips (Iwaki Glass Co., Ltd.) and immunostained with the following modifications: fixation, with 1% (w/v) formaldehyde in PBS for 15 min at room temperature followed by permeabilization with 0.2% (v/v) Triton X-100 for 15 min or with 100% methanol at −20 °C for 10 min; blocking, with 1% (w/v) BSA/PBS for 15 min; primary antibodies, for 1 h at room temperature; secondary antibodies for 30 min. DNA was also stained with 1 μg/ml DAPI.

Fluorescence images were obtained by confocal microscopy (LSM 510 META, Carl Zeiss) and equipped with a microscope (Axiovert 200 M, Carl Zeiss), a Plan Apochromat 40×/1.3 NA, 63×/1.4 NA, and 100×/1.4 NA oil immersion lens, a Plan Apochromat 150×/1.35 NA glycerol immersion lens, and LSM Image Browser software (Carl Zeiss).

Fig. 5*A* was obtained by using the DeltaVision system (Applied Precision), as described previously (32), equipped with a microscope (IX70, Olympus), a Plan Apochromat 100×/1.40 NA oil immersion lens (Olympus), and a cooled charge-coupled device camera (CoolSNAP HQ, Photometrics). The images were obtained with 0.2-μm intervals in a z section, deconvolved, and integrated with softWoRx software (Applied Precision).

##### FISH

Mouse eyes were fixed with 4% (w/v) paraformaldehyde in PBS overnight at 4 °C and embedded in paraffin. 5-μm-thick sections were prepared and mounted. Slides were deparaffinized and then treated with 0.1% (w/v) pepsin, 0.1 n HCl for 15 min. FISH probes were mounted with specimen, denatured for 10 min at 90 °C, and then hybridized overnight at 37 °C. After hybridization, slides were washed stringently in 50% formamide, 2× saline sodium citrate (SSC), and 1× SSC. DNA was also stained with 1 μg/ml DAPI.

Fluorescence image was captured with a fluorescence microscope (Leica CW-4000). The mouse two-color FISH probe (Chromosome 12 probe; labeled with Spectrum Green, chromosome 19 probe; labeled with Cy3) was purchased from Chromosome Science Labo Inc. (Sapporo, Japan).

For the cultured cells, some modifications were made. In brief, cells were fixed with Carnoy's fixative (3:1 methanol/glacial acetic acid) at room temperature for 5 min with three changes before FISH probes were hybridized to the samples.

##### Antibodies

The following primary antibodies were used: polyclonal guinea pig anti-vimentin (Progen; Germany); monoclonal rabbit anti-desmin (Y266, Abcam, Cambridge, UK); monoclonal mouse anti-GFAP (GA5, Cell Signaling Technology, Beverly, MA); monoclonal mouse anti-nestin (Millipore, Billerica, MA); polyclonal rabbit anti-HSP70 (D69; Cell Signaling Technology, Beverly; MA); HRP-conjugated polyclonal rabbit anti-α-tubulin (Abcam, Cambridge, UK); monoclonal rat anti E-cadherin (ECCD2, Cell Signaling Technology, Beverly, MA); monoclonal mouse anti β-catenin (Transduction Laboratories, Lexington, KY); polyclonal rabbit anti-AQP0/MIP (Alpha Diagnostic International, San Antonio, TX); polyclonal rabbit anti-γ-tubulin (ab11321, Abcam, Cambridge, UK); polyclonal goat anti-vimentin (25); monoclonal rabbit anti-vimentin (D21H3, Cell Signaling Technology, Beverly, MA); monoclonal mouse anti-α-tubulin (B-5-1-2, Sigma); polyclonal rabbit anti-PCNA (ab15497, Abcam, Cambridge, UK); and monoclonal anti-γ-tubulin (GTU-88, Sigma). Primary antibodies were detected using species-specific secondary antibodies conjugated to either Alexa Fluor 488 or 555 (Invitrogen).

##### Quantitative RT-PCR

Total RNA was prepared from eye lenses using TRIzol® (Invitrogen) in accordance with the manufacturer's instructions. Total RNA was reverse-transcribed using SuperScript®VILO^TM^ cDNA synthesis kit (Invitrogen). TaqMan analysis was performed. Primer sequences for p16^Ink4a^ and p19^Arf^ were as published (33). Other primers are the TaqMan®Gene Expression Assays by Applied Biosystems:E2f1 (Mm00432936_m1), p15 (Mm00483241_m1), p21 (Mm04205640_g1), GAPDH (Mm99999915_g1), and Ndrg2 (Mm00443481_g1).

##### Primary Cell Culture

Primary lens epithelial cell cultures were established by microdissecting the anterior lens capsule and then culturing on collagen I-coated plastic dish (BD Biosciences) with Dulbecco's modified Eagle's medium supplemented with 10% (v/v) FCS and antibiotics. Sequentially, they were replaced on collagen I-coated coverslips (Iwaki Glass Co., Ltd.) and cultured for a total of 7 days from the harvest, followed by the fixation.

##### Transmission Electron Microscopy of Lens Fiber Cells

The fixation was based on the following standard method with some modifications for the equatorial section. Lens was fixed with 2% fresh formaldehyde and 2.5% glutaraldehyde in 0.1 m sodium cacodylate buffer, pH 7.4, for 2 h at room temperature. After washing with 0.1 m cacodylate buffer, pH 7.4, they were postfixed with ice-cold 1% OsO_4_ in the same buffer for 2 h. The samples were rinsed with distilled water, stained with 0.5% aqueous uranyl acetate for 2 h or overnight at room temperature, dehydrated with ethanol and propylene oxide, and embedded in Poly/Bed 812 (Polysciences, Inc.). Then ultrathin sections were cut through the lens equator, double stained with uranyl acetate and Reynolds's lead citrate, and viewed with a transmission electron microscope (JEM-1010; JEOL) with a charge-coupled device camera (BioScan model 792; Gatan, Inc.) at an accelerating voltage of 100 kV.

##### Statistical Analyses

Mean values ± S.E. were calculated and used to test for significance between treatments in each dataset using the two-tailed Student's *t* test (Graph Pad software). Differences were considered significant when *, *p* < 0.05; **, *p* < 0.01; and ***, *p* < 0.001.

## RESULTS

### 

#### 

##### Lens Disorder in VIM^SA/SA^ Mice

**FIGURE 2.**
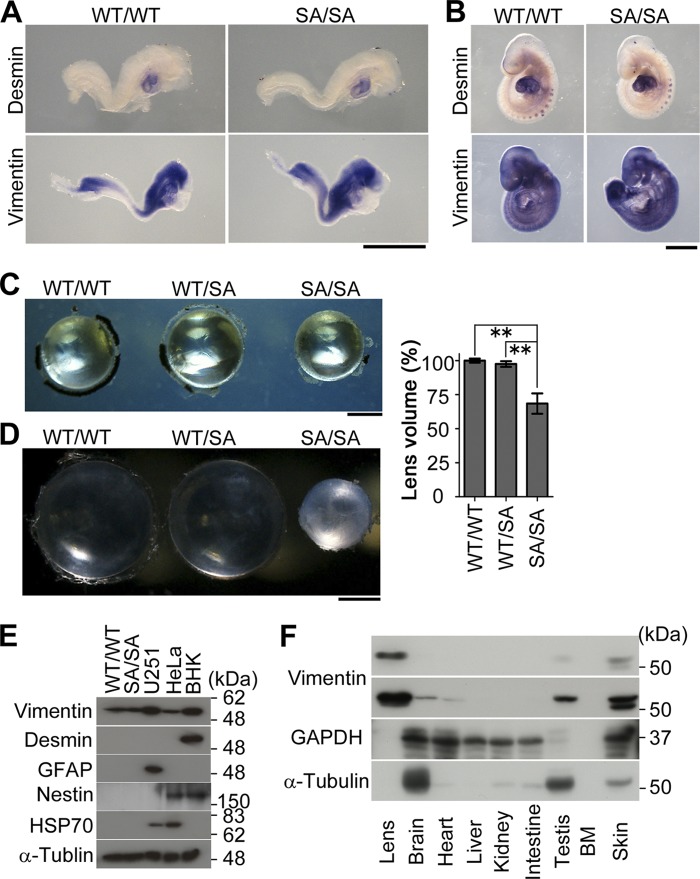
**Optical disorder in knock-in mice (*VIM^SA/SA^*).** *A* and *B, in situ* hybridization analyses of mouse whole embryos at E8.5 (*A*) and E9.5 (*B*) using vimentin and desmin probes. *C* and *D,* lens from 4-month-old (*C*) or 11-month-old (*D*) mice. *Bar graph* shows the quantification of the volume of lens, normalized to *VIM^WT/WT^* lens (*n* = 5 mice per genotype; *C*). The volume of 4-month-old lens (*C*) was calculated with the following mathematical formula: *V* = 4π*Rr*^2^/3 (*R*, major radius; *r,* minor axis). *E*, total lens lysates (20 μg) prepared from 4-month-old mice were analyzed by Western blotting. The lysates of U251, HeLa, and baby hamster kidney (*BHK*) cells were loaded as positive controls. *F*, vimentin expression pattern of indicated mouse tissues. 15 μg of total protein are applied in each lane. *Scale bars,* 500 μm (*A* and *B*) or 1 mm (*C* and *D*). **, *p* < 0.01.

Knock-in mice were generated that only expressed vimentin mutated at Ser-6, Ser-24, Ser-38, Ser-46, Ser-55, Ser-64, Ser-65, Ser-71, Ser-72, Ser-82, and Ser-86 to Ala (Fig. 1, *A–F*). These serine residues in mouse vimentin have been identified as phosphorylation sites during mitosis. Western blot analyses of embryos showed no significant difference in vimentin protein levels between WT (*VIM^WT/WT^*), heterozygous (*VIM^WT/SA^*), and homozygous (*VIM^SA/SA^*) mice (Fig. 1*F*). Next, we compared the expression patterns of vimentin and desmin in *VIM^WT/WT^* and *VIM^SA/SA^* embryos. There were only marginal differences in vimentin and desmin expression, as judged by *in situ* hybridization of mouse whole embryos at E8.5 (Fig. 2*A*) and E9.5 (Fig. 2*B*). These data collectively indicate the successful generation of vimentin mutant mice.

*VIM^SA/SA^* mice were viable, but the eyes were microophthalmic, and the lenses were smaller than those in littermate controls (Fig. 2*C*). The gross morphology and histology of the eye tissues were unchanged for *VIM^WT/WT^* and *VIM^WT/SA^* mice (Fig. 2*C*). Lens cataract was observed in *VIM^SA/SA^* mice at 11 months old, whereas *VIM^WT/WT^* and *VIM^WT/SA^* mice all had clear lenses (Fig. 2*D*). As shown in Fig. 2*E*, we observed no redundant protein expression of desmin, GFAP, and nestin (with which vimentin can form heteropolymeric filaments) (3) in *VIM^SA/SA^* lens. The fact that the lens is the tissue where vimentin is the most abundantly expressed (Fig. 2*F*) likely explains the cataract phenotype in *VIM^SA/SA^* mice.

##### Binucleation and Aneuploidy in VIM^SA/SA^ Lens

**FIGURE 3.**
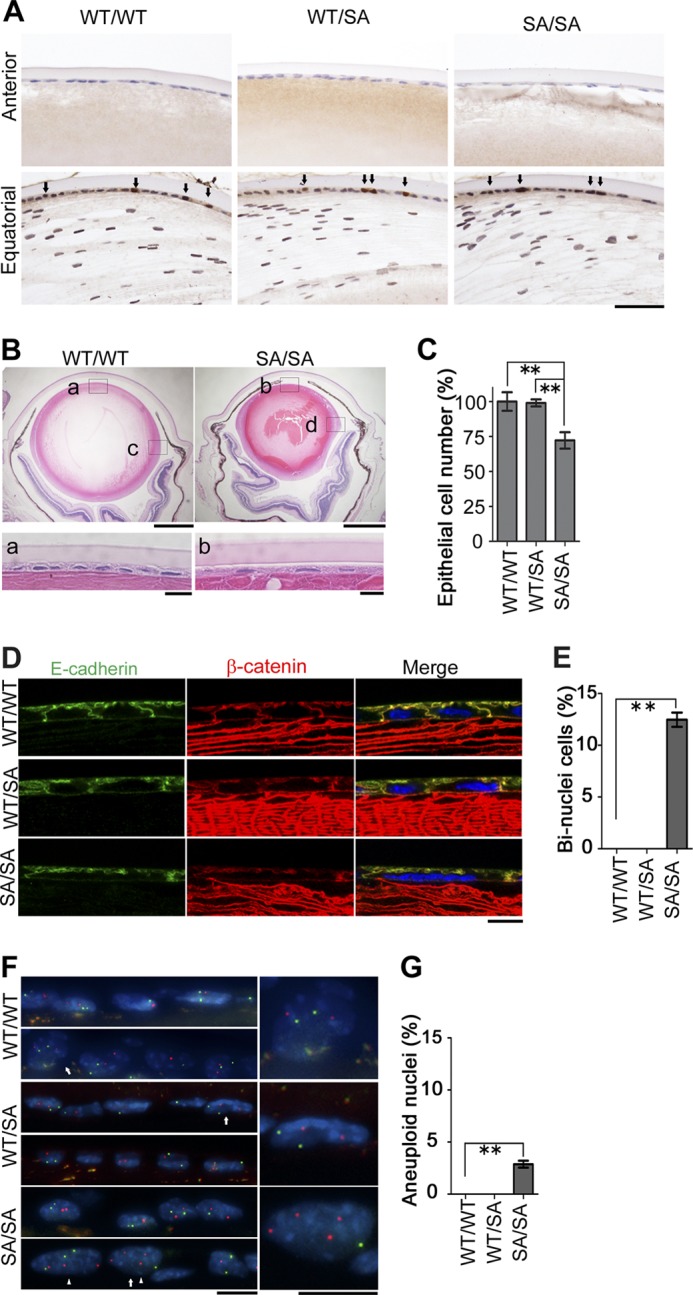
**Changes in the epithelial cells in the anterior region of *VIM^SA/SA^* lenses.** *A*, active dividing site of lens epithelium. Cells in lens tissue were stained with anti-PCNA. *Arrows* indicate PCNA-positive nuclei. *B,* microophthalmia in the *VIM^SA/SA^* mice. Lens from 4-month-old *VIM^WT/WT^* or *VIM^SA/SA^* mice was stained with H&E. Magnified images of anterior or equatorial regions of lens are also shown below (*a* and *b*) or in Fig. 4*A* (*c* and *d*), respectively. *C, bar graph* shows the cell number of epithelium per 500 × 100 μm area, normalized to *VIM^WT/WT^* mice (*n* = 3 mice per genotype); we observed at least 10 sections per each lens for the calculation. *D* and *E,* immunostaining (*D*) of lens epithelial cells with anti-E-cadherin (*green*), anti-β-catenin (*red*), and DAPI (*blue*; nuclei). *Bar graph* (*E*) shows the percentage of binucleate cells per 500 × 100 μm area; we observed at least 10 sections per each lens for the calculation (*n* = 3 mice per genotype). *E–G,* FISH analyses of nuclei in each genotype of mice. *Green* or *red* color represents mouse chromosome 12 or 19, respectively (*G*). Magnified images of nuclei indicated as *arrows* are shown at *right. Arrowheads* indicate aneuploid nuclei (*F*). Quantification of aneuploid nuclei is also shown (*G*; *n* = 3 mice per genotype). *Scale bars,* 50 μm (*A*), 500 μm (*B*, *upper*), 40 μm (*B*, *lower*), or 10 μm (*D* and *F*). **, *p* < 0.01.

A single layer of epithelial cells covers the anterior hemisphere of the lens. The cells in the central zone of the epithelium (anterior region) rarely undergo cell division, but at the lens equator there is a band of dividing cells termed the germinative zone in the epithelium (equatorial region). The progeny of these cells in the germinative zone of the equatorial region differentiate into lens fiber cells, a process that starts in the transitional zone of the equatorial region (34). The immunohistochemical analyses with anti-PCNA (which stains active DNA replication sites in cells; Fig. 3*A*) identified the germinative zone (34).

**FIGURE 4.**
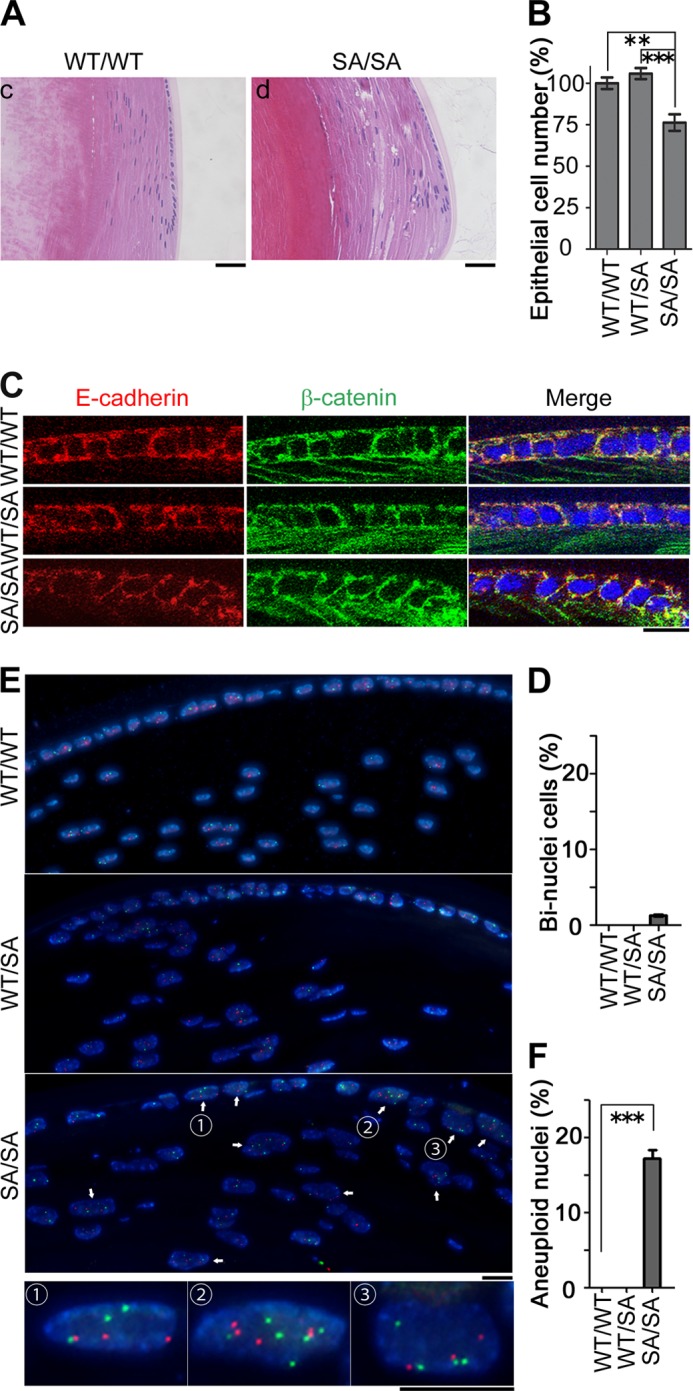
**Changes in the epithelial cells in the equatorial region of *VIM^SA/SA^* lenses.** *A–F,* histological analyses at equatorial portion of lens were performed according to the legend of Fig. 3. *Bar graphs* show the relative proportion of cell number or binuclear ratio per 300 × 100 μm area; we observed at least 10 sections per each lens for the calculation (*B* and *D*). *Scale bars,* 40 μm (*A*) or 10 μm (*C* and *E*). **, *p* < 0.01; ***, *p* < 0.001.

Histological changes were observed in both the anterior (Fig. 3, *B* and *C*) and equatorial regions (Fig. 4, *A* and *B*) of *VIM^SA/SA^* lenses. The number of lens epithelial cells was significantly decreased in *VIM^SA/SA^* mice, compared with *VIM^WT/WT^* or *VIM^WT/SA^* mice. In contrast, there was no significant difference between similar regions in *VIM^WT/WT^* and *VIM^WT/SA^* mice. Because binucleation (multinucleation) was observed in tissue culture cells expressing phosphorylation-compromised vimentin (21), lenses from *VIM^SA/SA^* were stained with E-cadherin, β-catenin (the markers of cell-cell boundaries), and DAPI (nucleus). In both anterior (Fig. 3, *D* and *E*) and equatorial (Fig. 4, *C* and *D*) regions of *VIM^WT/WT^* or *VIM^WT/SA^* lens epithelium, each cell had one nucleus. In contrast, binucleate (multinucleate) cells were observed in *VIM^SA/SA^* lens epithelium. Approximately 13 and 1% of cells each had two nuclei in the anterior (Fig. 3, *D* and *E*) and equatorial (Fig. 4, *C* and *D*) regions, respectively. FISH analyses using mouse chromosome 12 (*green*) and 19 (*red*) probes were used to determine chromosomal instability (CIN) in *VIM^SA/SA^* lens. Approximately 3 and 17% of nuclei in the anterior (Fig. 3, *F* and *G*) and equatorial (Fig. 4, *E* and *F*) regions, respectively, of *VIM^SA/SA^* lens epithelial cells had more than two FISH signals per chromosome. Aneuploid cells were not detected in *VIM^WT/WT^* or *VIM^WT/SA^* lens epithelial cells.

**FIGURE 5.**
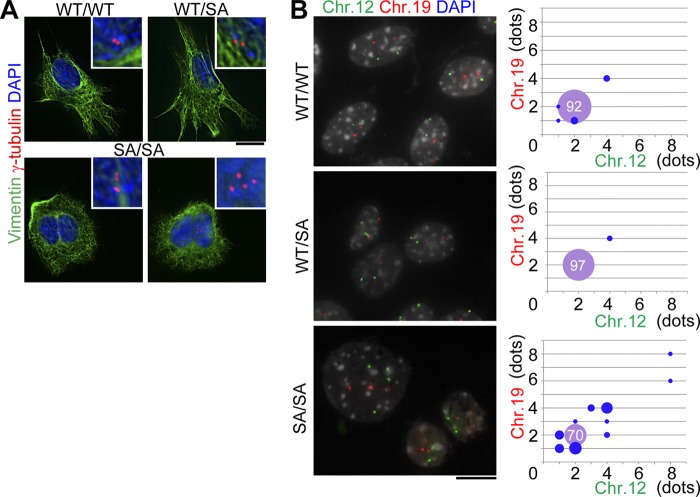
**Analysis of primary lens epithelial cell culture.** *A* and *B,* lens epithelial cells were primarily cultured for 7 days and then subjected to immunostaining (*A*) or FISH analyses (*B*). *Upper insets* indicate magnification images around centrosomes in each cell (*A*). Cells were classified as the numbers of chromosome (*Chr.*) 12 and 19 according to FISH data (*left photographs* in *B*). The proportion of each category is indicated as the *size of circle* (*right scattered graph* in *B*; *n* = 100 nuclei per genotype). The percentage of normal diploid (with each two chromosomes) is shown in the *violet circle* (*right graph* in *B*). *Scale bars,* 10 μm (*A* and *B*).

To analyze the mechanism by which CIN occurs in lens epithelial cells, we analyzed primary cultures of lens epithelial cells. *VIM^SA/SA^*-derived primary culture cells exhibited not only binucleation (Fig. 5*A*) but also aneuploidy (Fig. 5*B*). These phenomena were rarely observed in *VIM^WT/WT^*- or *VIM^WT/SA^*-derived primary culture cells. The binucleate cells in primary cell cultures derived from *VIM^SA/SA^* lenses possessed four centrosomes (Fig. 5*A*) consistent with cytokinetic failure causing binucleation (multinucleation) just like T24 cultured cells that express similar phosphorylation-compromised vimentin mutants (21). Does this account for aneuploidy in *VIM^SA/SA^* lens epithelial cells? Extra centrosomes have been reported to be sufficient to induce chromosome missegregation (such as lagging chromosomes) during cell division (35). Interestingly, tetraploid cells with four centrosomes showed a high frequency of chromosome missegregation whereas tetraploid cells with two centrosomes decreased to a level observed in diploid cells with two centrosomes (35). These observations led us to propose the following model. Lens epithelial cells in *VIM^SA/SA^* mice form an IF bridge, which induces binucleation (multinucleation) due to cytokinetic failure. The binucleation (multinucleation) results in abnormal numbers of centrosomes per cell, which in turn promotes aneuploidy. Given the regional differences in the frequency of cell division rates in the anterior and equatorial regions of the lens, this could explain why there is more binucleation (early event) in the central region and more aneuploidy (later event) in the equatorial zone (Fig. 3 *versus* Fig. 4).

##### Premature Aging in VIM^SA/SA^ Lens

How CIN induces microophthalmia and cataract in *VIM^SA/SA^* mice is an important question. As judged by TUNEL staining, it is less likely that CIN promotes apoptosis in *VIM^SA/SA^* lens.^4^ Table 1 summarizes the time course of the appearance of the lens abnormalities. Two months after birth, all lens abnormalities have started to appear in some *VIM^SA/SA^* mice. The abnormalities in lens size and chromosomal stability were observed in all *VIM^SA/SA^* mice by 3 months, whereas it took almost 1 year before all *VIM^SA/SA^* mice exhibited cataract phenotype. These observations suggested that lens cataract followed after CIN in *VIM^SA/SA^* lens.

**TABLE 1:** **Age-dependent lens abnormality in Vim^SA/SA^ mice**

	Abnormal lens size	Lens fiber disorientation	Bi nuclei	Aneuploidy	Cataract
**Homozygous (SA/SA)**					
2 months	*n* = 1/3	*n* = 1/3	*n* = 1/3	*n* = 1/3	*n* = 1/3
3 months	*n* = 4/4	*n* = 4/4	*n* = 4/4	*n* = 4/4	*n* = 1/4
4 months	*n* = 4/4	*n* = 4/4	*n* = 4/4	*n* = 4/4	*n* = 2/4
6 months	*n* = 4/4	*n* = 4/4	*n* = 4/4	*n* = 4/4	*n* = 2/4
8 months	*n* = 4/4	*n* = 4/4	*n* = 4/4	*n* = 4/4	*n* = 3/4
12 months	*n* = 3/3	*n* = 3/3	*n* = 3/3	*n* = 3/3	*n* = 3/3

**Heterozygous (WT/SA)**					
2 months	*n* = 0/3	*n* = 0/3	*n* = 0/3	*n* = 0/3	*n* = 0/3
3 months	*n* = 0/4	*n* = 0/4	*n* = 0/4	*n* = 0/4	*n* = 0/4
4 months	*n* = 0/4	*n* = 0/4	*n* = 0/4	*n* = 0/4	*n* = 0/4
6 months	*n* = 0/4	*n* = 0/4	*n* = 0/4	*n* = 0/4	*n* = 0/4
8 months	*n* = 0/4	*n* = 0/4	*n* = 0/4	*n* = 0/4	*n* = 0/4
12 months	*n* = 0/3	*n* = 0/3	*n* = 0/3	*n* = 0/3	*n* = 0/3

**Wild type (WT/WT**)					
2 months	*n* = 0/3	*n* = 0/3	*n* = 0/3	*n* = 0/3	*n* = 0/3
3 months	*n* = 0/4	*n* = 0/4	*n* = 0/4	*n* = 0/4	*n* = 0/4
4 months	*n* = 0/4	*n* = 0/4	*n* = 0/4	*n* = 0/4	*n* = 0/4
6 months	*n* = 0/4	*n* = 0/4	*n* = 0/4	*n* = 0/4	*n* = 0/4
8 months	*n* = 0/4	*n* = 0/4	*n* = 0/4	*n* = 0/4	*n* = 0/4
12 months	*n* = 0/3	*n* = 0/3	*n* = 0/3	*n* = 0/3	*n* = 0/3

**FIGURE 6.**
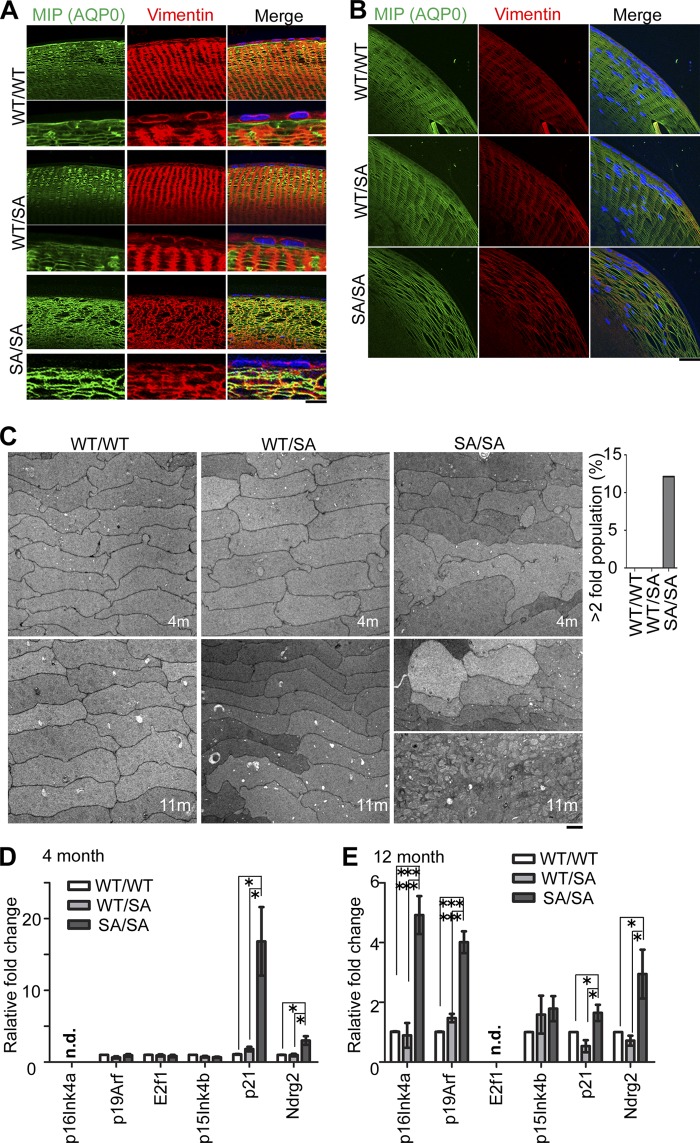
**Progeria-like phenotypes apparent in *VIM^SA/SA^* lenses.** *A* and *B*, disorganization of lens fiber cells in *VIM^SA/SA^* mice. Anterior (*A*) or equatorial (*B*) lens fiber cells from 4-month-old mice were immunostained with anti-MIP (AQP0; *green*), anti-vimentin (*red*), and DAPI (*blue*; nuclei). *C,* electron micrographs of lens at 4 (*upper*) or 11 (*lower*) months of age. The dispersion in the size distribution was evaluated as the percentage of cells >2 larger than the average size of cells (*right graph*). *D* and *E,* mRNA expression of the indicated genes were analyzed through quantitative RT-PCR using the lens of 4-month-old (*D*) or 12-month-old (*E*) mice (*n* = 5 mice per genotype). *n.d.* means “no detected signals.” *Scale bars,* 30 μm (*A*, *upper*) or 10 μm (*A* (*lower*) and *B* and *C*). *, *p* < 0.05; ***, *p* < 0.001.

To examine lens fiber morphology in detail, lens epithelial cells were costained with anti-AQP0 (also called membrane intrinsic protein (MIP)) and anti-vimentin. Lens fiber cells were disorganized at 4 months as seen by the AQP0 staining (Fig. 6, *A* and *B*) after birth when CIN was observed in all *VIM^SA/SA^* mice (also see Table 1). The vimentin network was also disorganized, but no vimentin aggregates were seen (Fig. 6, *A* and *B*).

Inter-fiber spaces were also increased in *VIM^SA/SA^* lens (Fig. 6, *A* and *B*), and in the bow region of the lens, the characteristic distribution of the fiber cell nuclei was disrupted (Figs. 4*A* and 6*B*). Vacuoles (area devoid of cellular materials; Fig. 4*A*) were also apparent in *VIM^SA/SA^* lenses. Such observations are consistent with other mouse models that develop lens cataract (36–39).

The cross-sectional profiles of fiber cells at the lens equator were analyzed by transmission electron microscopy (40). The profiles of both *VIM^WT/WT^* or *VIM^WT/SA^* lens fiber cells were very regular maintaining their characteristic hexagonal geometry over 11 months (Fig. 6*C*). *VIM^SA/SA^* lens fiber cells were, however, irregular both in size and shape. Size variation was very apparent as too was the disorganized membrane morphology manifested in, for example, irregular membrane processes (Fig. 6*C*). In samples from 11-month-old *VIM^SA/SA^* mice, the fiber cell boundaries were apparently lost in some areas (see a *right lowest photograph* in Fig. 6*C*), which we interpret as a degenerative process (41). Even with the increased resolution of the electron microscope, no electron dense aggregates were observed in the *VIM^SA/SA^* lenses. The observed changes in the plasma membranes of the *VIM^SA/SA^* lenses resemble those seen in age-related cataracts (41).

Given this comparison, real time PCR analyses to monitor mRNA levels of several senescence (aging)-related genes (p21, Ndrg2, p16^Ink4a^, and p19^Arf^) were performed on lens samples. The induction of p21 was recently reported to promote senescence in lens epithelial cells leading to cataract formation (42). N-myc downstream regulated gene 2 (Ndrg2) is considered to be a lens-specific senescent marker (43). Two aging biomarkers of various human and rodent tissues, p16^Ink4a^ and p19^Arf^ (44), were also reported to be induced in eye tissue of hypomorphic BubR1 (*BubR1^H/H^*) progeroid mice (45). The elevation of p21 promotes senescence of lens epithelial cells and cataract formation, whereas the later elevation of p16^Ink4a^ and p19^Arf^ is related to age-related tissue deterioration (42).

As shown in Fig. 6*D*, mRNA expression of p21 or Ndrg2 was significantly elevated in lens of 4-month-old *VIM^SA/SA^* mice, compared with the littermate control. 12 months after birth, transcript levels of not only p21 and Ndrg2 but also p16^Ink4a^ and p19^Arf^ were significantly higher in *VIM^SA/SA^* lens than in *VIM^WT/WT^* or *VIM^WT/SA^* lens (Fig. 6*E*). These data suggest that premature cataract formation might represent a progeroid-like lens phenotype in *VIM^SA/SA^* mice.

## DISCUSSION

In this study, we have analyzed the physiological effects of the expression of a vimentin mutant that will remain unphosphorylated during cell division by replacing with alanine all the serine residues known to be phosphorylated in mitosis. Binucleate (multinucleate) epithelial cells were observed in the lenses of *VIM^SA/SA^* mice (Figs. 3 and 4), mimicking the observations made with T24 cultured cells that expressed phosphorylation-compromised vimentin. Pairs of cells remained connected by an IF bridge, which subsequently induced cytokinetic failure (21). We also found aneuploid lens epithelial cells in *VIM^SA/SA^* mice (Figs. 3 and 4), which was unexpected. Perhaps this is due to the presence of additional centrosomes in the binucleated cells (as discussed above). CIN accelerates premature aging, and in *VIM^SA/SA^* mice this was manifested as cataracts, the classic age-related phenotype of the eye lens.

Congenital cataracts can be caused by vimentin mutations, for example, the E151K (*VIM^WT/E151K^*) mutation in the vimentin rod domain (46). Magin and co-workers (47) also reported lens cataract formation in the transgenic mice with vimentin carrying R113C mutation (VimR113C). The cataract pathology observed for both these mutations in the vimentin rod domain is different from that reported here in several respects. Both are autosomal dominant mutations (46, 47). In contrast, no cataract pathology was observed in *VIM^WT/SA^* mice. The inheritance is therefore recessive and not dominant in character. Rod domain vimentin mutants also formed protein aggregates, and the endogenous vimentin network was disrupted (46, 47). Such consequences were rarely, if at all, observed in both *VIM^WT/SA^* and *VIM^SA/SA^* mice (Fig. 6). This observation is consistent with our previous finding that SA mutants can normally form vimentin filament networks in interphase cells (25). Finally, the expression of HSP70 was elevated in response to abnormal vimentin structures such as protein aggregates in transgenic VimR113C mice (47), but such a phenomenon was not observed in the lenses of *VIM^SA/SA^* mice (Fig. 2*E*). Taken together, it is less likely that the expression of phosphorylation-compromised vimentin may induce abnormal vimentin filament structure and subsequently lens cataract.

In conclusion, this study documents for the first time the physiological importance of vimentin phosphorylation during cell division in the lens. It also paves the way for future studies evaluating the relationship between CIN and premature aging.

## Footnotes

4 H. Tanaka and M. Inagaki, unpublished observations.

## Footnotes

3 The abbreviations used are:

IF: intermediate filament
AQP0: aquaporin 0
CIN: chromosomal instability
GFAP: glial fibrillary acidic protein
MIP: membrane intrinsic protein
Ndrg2: N-myc downstream-regulated gene 2
PCNA: proliferating cell nuclear antigen
SA: substitution of serine residues to alanine on vimentin
*VIM*: vimentin.

## References

[1] Eriksson J. E. Opal P. Goldman R. D. (1992) Intermediate filament dynamics. Curr. Opin. Cell Biol. 4, 99–104 1558758 10.1016/0955-0674(92)90065-k

[2] Fuchs E. Weber K. (1994) Intermediate filaments: structure, dynamics, function, and disease. Annu. Rev. Biochem. 63, 345–382 7979242 10.1146/annurev.bi.63.070194.002021

[3] Eriksson J. E. Dechat T. Grin B. Helfand B. Mendez M. Pallari H. M. Goldman R. D. (2009) Introducing intermediate filaments: from discovery to disease. J. Clin. Invest. 119, 1763–1771 19587451 10.1172/JCI38339 PMC2701876

[4] Quinlan R. A. Brenner M. Goldman J. E. Messing A. (2007) GFAP and its role in Alexander disease. Exp. Cell Res. 313, 2077–2087 17498694 10.1016/j.yexcr.2007.04.004 PMC2702672

[5] Pekny M. Lane E. B. (2007) Intermediate filaments and stress. Exp. Cell Res. 313, 2244–2254 17524394 10.1016/j.yexcr.2007.04.023

[6] Capetanaki Y. Bloch R. J. Kouloumenta A. Mavroidis M. Psarras S. (2007) Muscle intermediate filaments and their links to membranes and membranous organelles. Exp. Cell Res. 313, 2063–2076 17509566 10.1016/j.yexcr.2007.03.033

[7] Song S. Landsbury A. Dahm R. Liu Y. Zhang Q. Quinlan R. A. (2009) Functions of the intermediate filament cytoskeleton in the eye lens. J. Clin. Invest. 119, 1837–1848 19587458 10.1172/JCI38277 PMC2701874

[8] Ivaska J. Pallari H. M. Nevo J. Eriksson J. E. (2007) Novel functions of vimentin in cell adhesion, migration, and signaling. Exp. Cell Res. 313, 2050–2062 17512929 10.1016/j.yexcr.2007.03.040

[9] Inagaki M. Matsuoka Y. Tsujimura K. Ando S. Tokui T. Takahashi T. Inagaki N. (1996) Dynamic property of intermediate filaments: regulation by phosphorylation. BioEssays 18, 481–487

[10] Sihag R. K. Inagaki M. Yamaguchi T. Shea T. B. Pant H. C. (2007) Role of phosphorylation on the structural dynamics and function of types III and IV intermediate filaments. Exp. Cell Res. 313, 2098–2109 17498690 10.1016/j.yexcr.2007.04.010 PMC2570114

[11] Inagaki M. Nishi Y. Nishizawa K. Matsuyama M. Sato C. (1987) Site-specific phosphorylation induces disassembly of vimentin filaments *in vitro*. Nature 328, 649–652 3039376 10.1038/328649a0

[12] Goto H. Inagaki M. (2007) Production of a site- and phosphorylation state-specific antibody. Nat. Protoc. 2, 2574–2581 17948000 10.1038/nprot.2007.374

[13] Nishizawa K. Yano T. Shibata M. Ando S. Saga S. Takahashi T. Inagaki M. (1991) Specific localization of phosphointermediate filament protein in the constricted area of dividing cells. J. Biol. Chem. 266, 3074–3079 1993680

[14] Goto H. Kosako H. Inagaki M. (2000) Regulation of intermediate filament organization during cytokinesis: possible roles of Rho-associated kinase. Microsc. Res. Tech. 49, 173–182 10816257 10.1002/(SICI)1097-0029(20000415)49:2<173::AID-JEMT10>3.0.CO;2-A

[15] Izawa I. Inagaki M. (2006) Regulatory mechanisms and functions of intermediate filaments: a study using site- and phosphorylation state-specific antibodies. Cancer Sci. 97, 167–174 16542212 10.1111/j.1349-7006.2006.00161.x PMC11159468

[16] Matsuoka Y. Nishizawa K. Yano T. Shibata M. Ando S. Takahashi T. Inagaki M. (1992) Two different protein kinases act on a different time schedule as glial filament kinases during mitosis. EMBO J. 11, 2895–2902 1379174 10.1002/j.1460-2075.1992.tb05358.x PMC556770

[17] Goto H. Yasui Y. Kawajiri A. Nigg E. A. Terada Y. Tatsuka M. Nagata K. Inagaki M. (2003) Aurora-B regulates the cleavage furrow-specific vimentin phosphorylation in the cytokinetic process. J. Biol. Chem. 278, 8526–8530 12458200 10.1074/jbc.M210892200

[18] Kawajiri A. Yasui Y. Goto H. Tatsuka M. Takahashi M. Nagata K. Inagaki M. (2003) Functional significance of the specific sites phosphorylated in desmin at cleavage furrow: Aurora-B may phosphorylate and regulate type III intermediate filaments during cytokinesis coordinatedly with Rho-kinase. Mol. Biol. Cell 14, 1489–1500 12686604 10.1091/mbc.E02-09-0612 PMC153117

[19] Tsujimura K. Ogawara M. Takeuchi Y. Imajoh-Ohmi S. Ha M. H. Inagaki M. (1994) Visualization and function of vimentin phosphorylation by cdc2 kinase during mitosis. J. Biol. Chem. 269, 31097–31106 7983050

[20] Chou Y. H. Bischoff J. R. Beach D. Goldman R. D. (1990) Intermediate filament reorganization during mitosis is mediated by p34cdc2 phosphorylation of vimentin. Cell 62, 1063–1071 2169348 10.1016/0092-8674(90)90384-q

[21] Yamaguchi T. Goto H. Yokoyama T. Silljé H. Hanisch A. Uldschmid A. Takai Y. Oguri T. Nigg E. A. Inagaki M. (2005) Phosphorylation by Cdk1 induces Plk1-mediated vimentin phosphorylation during mitosis. J. Cell Biol. 171, 431–436 16260496 10.1083/jcb.200504091 PMC2171270

[22] Goto H. Kosako H. Tanabe K. Yanagida M. Sakurai M. Amano M. Kaibuchi K. Inagaki M. (1998) Phosphorylation of vimentin by Rho-associated kinase at a unique amino-terminal site that is specifically phosphorylated during cytokinesis. J. Biol. Chem. 273, 11728–11736 9565595 10.1074/jbc.273.19.11728

[23] Kosako H. Amano M. Yanagida M. Tanabe K. Nishi Y. Kaibuchi K. Inagaki M. (1997) Phosphorylation of glial fibrillary acidic protein at the same sites by cleavage furrow kinase and Rho-associated kinase. J. Biol. Chem. 272, 10333–10336 9099667 10.1074/jbc.272.16.10333

[24] Inada H. Togashi H. Nakamura Y. Kaibuchi K. Nagata K. Inagaki M. (1999) Balance between activities of Rho kinase and type 1 protein phosphatase modulates turnover of phosphorylation and dynamics of desmin/vimentin filaments. J. Biol. Chem. 274, 34932–34939 10574968 10.1074/jbc.274.49.34932

[25] Yasui Y. Goto H. Matsui S. Manser E. Lim L. Nagata Ki Inagaki M. (2001) Protein kinases required for segregation of vimentin filaments in mitotic process. Oncogene 20, 2868–2876 11420699 10.1038/sj.onc.1204407

[26] Yasui Y. Amano M. Nagata K. Inagaki N. Nakamura H. Saya H. Kaibuchi K. Inagaki M. (1998) Roles of Rho-associated kinase in cytokinesis; mutations in Rho-associated kinase phosphorylation sites impair cytokinetic segregation of glial filaments. J. Cell Biol. 143, 1249–1258 9832553 10.1083/jcb.143.5.1249 PMC2133074

[27] O'Connell C. B. Wheatley S. P. Ahmed S. Wang Y. L. (1999) The small GTP-binding protein Rho regulates cortical activities in cultured cells during division. J. Cell Biol. 144, 305–313 9922456 10.1083/jcb.144.2.305 PMC2132903

[28] Niwa H. Masui S. Chambers I. Smith A. G. Miyazaki J. (2002) Phenotypic complementation establishes requirements for specific POU domain and generic transactivation function of Oct-3/4 in embryonic stem cells. Mol. Cell. Biol. 22, 1526–1536 11839818 10.1128/mcb.22.5.1526-1536.2002 PMC134688

[29] Ogawa K. Matsui H. Ohtsuka S. Niwa H. (2004) A novel mechanism for regulating clonal propagation of mouse ES cells. Genes Cells 9, 471–477 15147275 10.1111/j.1356-9597.2004.00736.x

[30] Tanaka H. Tamura A. Sekai M. Hamazaki Y. Minato N. (2011) Increased c-Myc activity and DNA damage in hematopoietic progenitors precede myeloproliferative disease in Spa-1-deficiency. Cancer Sci. 102, 784–791 21205094 10.1111/j.1349-7006.2011.01850.x

[31] Wilkinson D. G. (ed) (1992) In Situ Hybridization: A Practical Approach, pp. 75–83, IRL Press at Oxford University Press, Oxford

[32] Inoko A. Matsuyama M. Goto H. Ohmuro-Matsuyama Y. Hayashi Y. Enomoto M. Ibi M. Urano T. Yonemura S. Kiyono T. Izawa I. Inagaki M. (2012) Trichoplein and Aurora A block aberrant primary cilia assembly in proliferating cells. J. Cell Biol. 197, 391–405 22529102 10.1083/jcb.201106101 PMC3341160

[33] Krishnamurthy J. Ramsey M. R. Ligon K. L. Torrice C. Koh A. Bonner-Weir S. Sharpless N. E. (2006) p16INK4a induces an age-dependent decline in islet regenerative potential. Nature 443, 453–457 16957737 10.1038/nature05092

[34] Sharma K. K. Santhoshkumar P. (2009) Lens aging: effects of crystallins. Biochim. Biophys. Acta 1790, 1095–1108 19463898 10.1016/j.bbagen.2009.05.008 PMC2743770

[35] Ganem N. J. Godinho S. A. Pellman D. (2009) A mechanism linking extra centrosomes to chromosomal instability. Nature 460, 278–282 19506557 10.1038/nature08136 PMC2743290

[36] Boyle D. L. Takemoto L. Brady J. P. Wawrousek E. F. (2003) Morphological characterization of the αA- and αB-crystallin double knockout mouse lens. BMC Ophthalmol. 3, 3 12546709 10.1186/1471-2415-3-3 PMC149350

[37] Liu H. Du X. Wang M. Huang Q. Ding L. McDonald H. W. Yates J. R. 3rd Beutler B. Horwitz J. Gong X. (2005) Crystallin γB-I4F mutant protein binds to α-crystallin and affects lens transparency. J. Biol. Chem. 280, 25071–25078 15878859 10.1074/jbc.M502490200

[38] Bassuk J. A. Birkebak T. Rothmier J. D. Clark J. M. Bradshaw A. Muchowski P. J. Howe C. C. Clark J. I. Sage E. H. (1999) Disruption of the Sparc locus in mice alters the differentiation of lenticular epithelial cells and leads to cataract formation. Exp. Eye Res. 68, 321–331 10079140 10.1006/exer.1998.0608

[39] Chang B. Wang X. Hawes N. L. Ojakian R. Davisson M. T. Lo W. K. Gong X. (2002) A Gja8 (Cx50) point mutation causes an alteration of α3 connexin (Cx46) in semi-dominant cataracts of Lop10 mice. Hum. Mol. Genet. 11, 507–513 11875045 10.1093/hmg/11.5.507

[40] Nowak R. B. Fischer R. S. Zoltoski R. K. Kuszak J. R. Fowler V. M. (2009) Tropomodulin1 is required for membrane skeleton organization and hexagonal geometry of fiber cells in the mouse lens. J. Cell Biol. 186, 915–928 19752024 10.1083/jcb.200905065 PMC2753162

[41] Michael R. Bron A. J. (2011) The ageing lens and cataract: a model of normal and pathological ageing. Philos. Trans. R. Soc. Lond. B Biol. Sci. 366, 1278–1292 21402586 10.1098/rstb.2010.0300 PMC3061107

[42] Baker D. J. Weaver R. L. van Deursen J. M. (2013) p21 both attenuates and drives senescence and aging in BubR1 progeroid mice. Cell Rep. 3, 1164–1174 23602569 10.1016/j.celrep.2013.03.028 PMC3785294

[43] Zhang Z. F. Zhang J. Hui Y. N. Zheng M. H. Liu X. P. Kador P. F. Wang Y. S. Yao L. B. Zhou J. (2011) Up-regulation of NDRG2 in senescent lens epithelial cells contributes to age-related cataract in human. PLoS One 6, e26102 22043305 10.1371/journal.pone.0026102 PMC3197158

[44] Krishnamurthy J. Torrice C. Ramsey M. R. Kovalev G. I. Al-Regaiey K. Su L. Sharpless N. E. (2004) Ink4a/Arf expression is a biomarker of aging. J. Clin. Invest. 114, 1299–1307 15520862 10.1172/JCI22475 PMC524230

[45] Baker D. J. Perez-Terzic C. Jin F. Pitel K. S. Pitel K. Niederländer N. J. Jeganathan K. Yamada S. Reyes S. Rowe L. Hiddinga H. J. Eberhardt N. L. Terzic A. van Deursen J. M. (2008) Opposing roles for p16Ink4a and p19Arf in senescence and ageing caused by BubR1 insufficiency. Nat. Cell Biol. 10, 825–836 18516091 10.1038/ncb1744 PMC2594014

[46] Müller M. Bhattacharya S. S. Moore T. Prescott Q. Wedig T. Herrmann H. Magin T. M. (2009) Dominant cataract formation in association with a vimentin assembly disrupting mutation. Hum. Mol. Genet. 18, 1052–1057 19126778 10.1093/hmg/ddn440

[47] Bornheim R. Müller M. Reuter U. Herrmann H. Büssow H. Magin T. M. (2008) A dominant vimentin mutant upregulates Hsp70 and the activity of the ubiquitin-proteasome system, and causes posterior cataracts in transgenic mice. J. Cell Sci. 121, 3737–3746 18940912 10.1242/jcs.030312

